# The Impact of Hypertension on Cognitive Decline and Alzheimer's Disease and Its Management: A Systematic Review

**DOI:** 10.7759/cureus.65194

**Published:** 2024-07-23

**Authors:** Adam D Khan, Sara Elnagar, Mohamed Eltayeb, Shariq K Baluch, Ajay Kumar, Madhurta Kumari, Muskan Kumari, Muhammad Usman Fareed, Abdur Rehman, Abdullah Shehryar

**Affiliations:** 1 Internal Medicine, Frontier Medical & Dental College, Abbottabad, PAK; 2 Internal Medicine, NewYork-Presbyterian Queens Hospital, New York City, USA; 3 Internal Medicine, Namerah General Hospital, Namerah, SAU; 4 Internal Medicine, Universidad Autónoma de Guadalajara, Guadalajara, MEX; 5 Internal Medicine, Chandka Medical College, Larkana, PAK; 6 Surgery, Nishtar Medical University, Multan, PAK; 7 Surgery, Mayo Hospital, Lahore, PAK; 8 Internal Medicine, Allama Iqbal Medical College, Lahore, PAK

**Keywords:** blood pressure variability, randomized controlled trials, intervention strategies, blood pressure management, alzheimer's disease, cognitive decline, hypertension

## Abstract

Hypertension, a major risk factor for cardiovascular diseases, has also been linked to cognitive decline and Alzheimer's disease (AD). This systematic review synthesizes the current evidence on how managing hypertension may influence cognitive health, particularly among elderly populations and those with cognitive impairments. By analyzing data from randomized controlled trials (RCTs), clinical trials, and cross-sectional studies, we evaluated the efficacy of various interventions, including pharmacological treatments, lifestyle modifications, and multidomain approaches that address blood pressure (BP) variability and intensive versus standard blood pressure control. Our findings reveal that effective blood pressure management can mitigate cognitive decline and potentially alter the course of Alzheimer's disease. However, the results also highlight complexities, such as the risk of adverse effects from intensive blood pressure control on cognitive processing and hippocampal volume. This review underscores the need for tailored hypertension management strategies that balance cardiovascular health with cognitive outcomes, suggesting that stabilizing blood pressure variability could play a crucial role. Future research should focus on longitudinal studies to refine these management strategies and enhance treatment guidelines, improving overall outcomes for patients at risk of cognitive decline.

## Introduction and background

Hypertension, defined by the Joint National Committee as a consistently elevated blood pressure exceeding 140/90 mmHg, is a significant global health issue and a principal risk factor for cardiovascular diseases. Emerging research has broadened our understanding of its impact, linking hypertension to neurological disorders, notably cognitive decline and Alzheimer's disease (AD) [1]. These health issues pose major public challenges, adversely affecting life quality, driving up healthcare expenses, and increasing demands on caregivers. The pathogenesis of Alzheimer's disease is multifactorial, involving genetic predispositions and environmental interactions, with hypertension identified as a crucial, controllable risk factor [2]. Managing hypertension through lifestyle modifications and pharmacological interventions offers promising strategies for mitigating these effects [3].

The interplay between hypertension and cognitive impairment involves complex pathophysiological pathways [4]. Chronic hypertension can induce microvascular damage within the brain, disrupting cerebral blood flow and fostering the accumulation of amyloid plaques, a hallmark of Alzheimer's pathology. Additionally, it is associated with increased neuroinflammation and oxidative stress, which are pivotal in cognitive decline [5]. Unlike Alzheimer's disease, which primarily involves neurodegenerative processes, vascular dementia arises from these vascular abnormalities directly impairing cerebral blood flow, leading to brain ischemia and consequent cognitive deficits. Given the aging global population and the escalating prevalence of both hypertension and dementia, it is imperative to understand how effective blood pressure management might mitigate or postpone the onset of cognitive decline and Alzheimer's disease while also considering the distinct pathologies of vascular dementia [6].

This systematic review aims to assess the impact of hypertension management on cognitive decline and Alzheimer's disease by analyzing a variety of intervention strategies. We will synthesize data from diverse study designs, ranging from randomized controlled trials (RCTs) to cross-sectional studies, targeting elderly populations at risk for dementia, Alzheimer's patients, and hypertensive adults with cognitive impairments. This review focuses on evaluating the efficacy of various blood pressure management approaches, including multidomain interventions, the role of blood pressure variability, and the effects of intensive versus standard blood pressure control. Additionally, we will consider the influence of polypharmacy and physical activity. Our goal is to offer a comprehensive understanding of how tailored hypertension management can mitigate cognitive decline and alter the course of Alzheimer's disease by examining cognitive function, brain morphology, and related cerebrovascular and cardiovascular factors.

## Review

Materials and methods

Search Strategy

Our search methodology was meticulously crafted to align with the Preferred Reporting Items for Systematic Reviews and Meta-Analyses (PRISMA) guidelines, focusing on studies exploring the nexus between hypertension, blood pressure management, and cognitive outcomes, including Alzheimer's disease. Extensive searches were executed across key electronic databases such as PubMed, Medline, Embase, the Cochrane Library, and PsycINFO, covering publications from the inception of each database through June 2024.

The search utilized a blend of keywords and Medical Subject Headings (MeSH) relevant to our study aims, including terms such as "hypertension," "blood pressure management," "cognitive decline," "Alzheimer's disease," "intervention studies," and "randomized controlled trials." Boolean operators such as "AND" and "OR" were employed to strategically combine these terms, with search strings such as "hypertension AND cognitive decline AND Alzheimer's disease" and "blood pressure management AND intervention outcomes." To broaden the scope of our literature review, we examined the reference lists of identified articles and included clinical trial registries and relevant conference proceedings to encompass unpublished or ongoing research in this domain. An expert in medical literature, specializing in cardiovascular and neurological studies, evaluated our search strategy to ensure comprehensive and accurate coverage. The search was confined to peer-reviewed articles published in English, targeting clinical trials, longitudinal studies, and randomized controlled trials that investigate the impact of hypertension management strategies on cognitive health in populations at risk or already diagnosed with Alzheimer's disease.

Eligibility Criteria

The eligibility criteria for this systematic review are designed to ensure rigor and relevance. Our focus is on peer-reviewed research articles, including clinical trials, randomized controlled trials (RCTs), and longitudinal studies, that examine the relationship between hypertension management and cognitive outcomes, specifically cognitive decline and Alzheimer's disease. Eligible studies must be peer-reviewed and include clinical trials, RCTs, and longitudinal studies. The participant pool should consist of adults diagnosed with hypertension or at risk for cognitive decline and Alzheimer’s disease. Interventions should focus on hypertension management through pharmacological treatments, lifestyle adjustments, and multidomain approaches addressing blood pressure variability. Selected studies must gauge cognitive outcomes, including cognitive decline, Alzheimer's disease progression, or brain structure changes related to cognitive health. Only studies published in English from the inception of the databases until June 2024 are considered to ensure the inclusion of the latest findings.

To maintain the review's focus and integrity, certain criteria are used to exclude studies. Investigations not directly probing the impact of hypertension management on cognitive decline or Alzheimer's disease are omitted. Research conducted solely on animal models is disregarded to prioritize findings directly applicable to human patients. Grey literature sources, such as conference abstracts and unpublished studies, are excluded to uphold scientific rigor. Studies not published in English are omitted due to potential translation and interpretation challenges, which could compromise data reliability. Additionally, studies lacking sufficient detail regarding intervention methods or cognitive outcomes pertinent to hypertension management are excluded to ensure analytical depth and integrity.

Data Extraction

Our data extraction methodology was meticulously developed to ensure both the reliability and validity of the data gathered for our systematic review, which investigates the effects of hypertension and blood pressure management on cognitive decline and Alzheimer's disease. Initially, we conducted a preliminary screening of articles by assessing their titles and abstracts. Two independent reviewers assessed these documents to determine their relevance, categorizing them as "relevant," "not relevant," or "possibly relevant." This initial filtering was crucial to narrow down the focus to the articles most relevant to our review's objectives.

Following this preliminary assessment, articles identified as potentially relevant were subjected to a detailed full-text review. To promote consistency and precision in our data collection process, we utilized a standardized data extraction form designed in Microsoft Excel (Microsoft® Corp., Redmond, WA). Each reviewer independently populated this form, applying the pre-established inclusion and exclusion criteria to each article. If any discrepancies or disagreements arose during this phase, a third reviewer was consulted to mediate and ensure resolution through discussion, thus preserving the integrity and consistency of our data selection process.

The design of the data extraction form was carefully thought out to encapsulate crucial information vital for fulfilling the objectives of our review. The form included fields for the lead author's name, publication year, study type, study population, intervention details, study duration, primary and secondary outcomes, and any limitations noted within the study. This structured data extraction process allowed for an exhaustive analysis of each study, ensuring comprehensive consideration and integration of all pertinent data into our systematic review.

Data Analysis and Synthesis

Given the heterogeneity of the included studies in terms of design, populations, and outcomes, we conducted a qualitative synthesis rather than a meta-analysis. Each study was analyzed for key variables such as intervention type, population characteristics, and cognitive outcomes. We employed a narrative approach to synthesize the data, which allowed us to integrate diverse findings and identify overarching themes. This approach highlighted two primary insights: the impact of blood pressure control strategies, where intensive control showed not only potential cognitive benefits but also risks such as hippocampal volume reduction, and the effectiveness of multidomain interventions, which combined lifestyle changes and pharmacological treatments to improve cognitive outcomes.

The synthesis revealed that managing blood pressure variability could significantly influence cognitive health, with studies suggesting that minimizing variability might help mitigate cognitive decline, especially in Alzheimer's patients. These findings suggest a need for tailored hypertension management strategies that consider individual risk factors and health profiles, indicating directions for future research to optimize treatment guidelines and improve cognitive health outcomes in hypertensive patients.

Results

Study Selection Process

**Figure 1:**
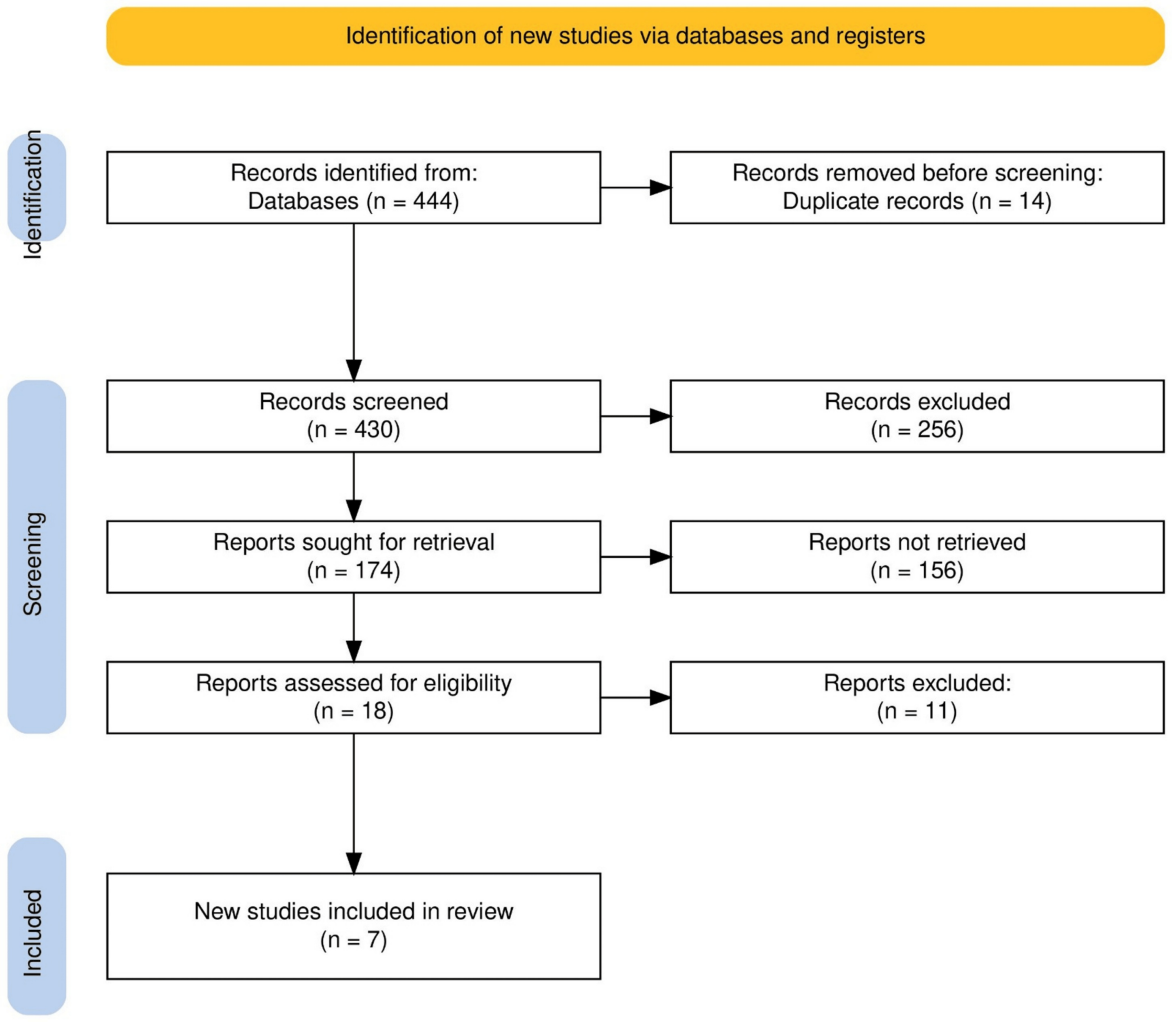
PRISMA flow diagram of the selection of studies for inclusion in the systematic review PRISMA: Preferred Reporting Items for Systematic Reviews and Meta-Analyses

The search across several databases and registers yielded a total of 444 records. After removing 14 duplicates, 430 records were screened. From these, 174 reports were identified for further assessment, leading to the retrieval of detailed reports. Of these, 18 were assessed for eligibility based on our inclusion criteria. Following a thorough review, 11 reports were excluded for failing to meet all criteria, leaving seven new studies that were included in the systematic review. The PRISMA flowchart provided in Figure 1 visualizes this study selection process clearly and methodically, ensuring transparency and adherence to the systematic review protocol.

Characteristics of Selected Studies

Our systematic review synthesized findings from seven studies on the effects of hypertension management on cognitive decline and Alzheimer's disease, covering varied interventions and populations. The study by Rosenberg et al. [7] showed that a multidomain intervention benefited 1,260 elderly at risk for dementia. The trial by de Heus et al. [8] with 460 Alzheimer's patients linked higher blood pressure variability to more significant cognitive and functional decline. The research of Nasrallah et al. [9] on 1,267 hypertensive adults found that intensive blood pressure control might adversely affect hippocampal volume. Similarly, the study by Rapp et al. [10] on 2,921 hypertensive adults observed that intensive treatment strategies did not improve memory and led to a decline in processing speed. Vidoni et al. [11] highlighted high polypharmacy prevalence in 514 older adults with hypertension, especially among those with coexisting conditions. Hajjar et al. [12] explored the cognitive benefits of antihypertensive drugs in 100 older adults. The trial by Cyarto et al. [13] on 156 older adults assessed physical activity's impact on the white matter changes in the brain, potentially slowing progression in those with mild cognitive impairments. These studies underscore the nuanced impacts of blood pressure management on cognitive health and advocate for tailored treatment strategies to optimize patient outcomes. A summary is provided in Table 1.

**Table 1:** Studies included in the systematic review RCT: randomized controlled trial, BP: blood pressure, MRI: magnetic resonance imaging, MCI: mild cognitive impairment, WMH: white matter hyperintensities

Lead author	Study type	Study population	Intervention	Duration	Primary outcome(s)	Method	Key measures	Results	Conclusion
Rosenberg et al. (2018) [7]	RCT	1,260 elderly at risk for dementia	Multidomain intervention versus regular advice	2 years	Change in cognition	Participants randomized 1:1, mixed-model analyses	Sociodemographics, cognition, cardiovascular factors	No modifiers of intervention effect	Intervention is beneficial regardless of participant characteristics
de Heus et al. (2019) [8]	Clinical trial	460 Alzheimer's patients	Assessment of blood pressure variability	1-1.5 years	Change in Alzheimer's disease severity	≥3 office BP measurements, variation independent of mean	BP variability, cognitive/functional scales	Higher BP variability is associated with more cognitive/functional decline	Targeting BP variability may slow Alzheimer's progression
Nasrallah et al. (2021) [9]	RCT secondary analysis	1,267 hypertensive adults	Intensive versus standard blood pressure control	4 years	Changes in brain MRI biomarkers	Brain MRI at baseline and follow-up	Hippocampal volume, atrophy, blood flow, white matter integrity	Intensive treatment associated with greater hippocampal volume decrease	Intensive BP control may adversely affect the hippocampus
Rapp et al. (2020) [10]	RCT substudy	2,921 hypertensive adults	Intensive versus standard blood pressure control	4.1 years	Memory and processing speed	Cognitive testing at baseline and biennially	Memory and processing speed composites	No difference in memory, more processing speed decline with intensive treatment	No clinically relevant cognitive effect of intensive BP control
Vidoni et al. (2020) [11]	RCT cross-sectional	514 older adults with hypertension	Review of medications, assessment of polypharmacy	Cross-sectional	Prevalence of polypharmacy	Structured medication interview	Number of medications	79.2% had polypharmacy, higher with diabetes/hyperlipidemia	High polypharmacy in this population
Hajjar et al. (2012) [12]	RCT	100 older adults with hypertension and cognitive impairment	Lisinopril, candesartan, or hydrochlorothiazide	1 year	Changes in cognition, cerebral blood flow, endothelial function	Double-blind RCT with cognitive, cerebrovascular assessments	Cognitive tests, cerebral blood flow, endothelial function	Not specified (study design paper)	Explore the impact of renin-angiotensin drugs on cognition and cerebrovasculature
Cyarto et al. (2013) [13]	RCT	156 older adults with memory complaints or MCI and vascular risk	Physical activity program	24 months	Change in white matter hyperintensities on MRI	Single-blind RCT with MRI, cognitive, and biomarker assessments	WMH on MRI, cognition, fitness, biomarkers	Not specified (study protocol)	Determine if physical activity slows WMH progression in at-risk older adults

Discussion

Our systematic review evaluated the impact of hypertension management on cognitive decline and Alzheimer's disease, emphasizing the influence of various interventions. Studies such as those by Hajjar et al. [12] highlighted the potential neuroprotective effects of renin angiotensin system (RAS) blockers such as lisinopril and candesartan. These medications not only manage blood pressure but also improve cerebral blood flow and endothelial function, suggesting a protective role against cognitive decline.

Lifestyle modifications, including physical activity and dietary adjustments, have shown positive effects on cognitive health. Such interventions, as described in the study by Cyarto et al. [13], enhance vascular health and reduce risk factors associated with cognitive deterioration, demonstrating their essential role in comprehensive hypertension management.

The effectiveness of combining pharmacological treatment with lifestyle changes was evident in the multidomain approach studied by Rosenberg et al. [7]. This synergistic effect points to improved cognitive outcomes, emphasizing the need for integrated treatment strategies. The association between blood pressure variability and cognitive outcomes was particularly noted in the study by de Heus et al. [8]. The findings suggest that minimizing blood pressure fluctuations is crucial in managing cognitive decline, especially in Alzheimer's patients.

The comparison of intensive and standard blood pressure control brought mixed results. While intensive strategies aimed to provide significant protection against cognitive decline, studies by Nasrallah et al. [9] and Rapp et al. [10] indicated potential adverse impacts on hippocampal volume and cognitive processing speed, underscoring the necessity for balanced and patient-specific treatment approaches.

Our review's findings align well with existing literature on the effects of hypertension management on cognitive outcomes, underscoring the significance of blood pressure control in mitigating cognitive decline and Alzheimer's disease progression. Landmark studies, such as those by the SPRINT MIND researchers [14], have similarly demonstrated that intensive blood pressure management can reduce the risk of mild cognitive impairment, a precursor to dementia. However, our review extends these findings by highlighting the complexities and potential drawbacks of intensive blood pressure interventions, such as the increased hippocampal atrophy reported by Nasrallah et al. [9] and the mixed outcomes on cognitive processing speeds observed by Rapp et al. [10]. These nuances contribute to a more balanced understanding of the role of blood pressure management in cognitive health.

Moreover, our review contributes significantly to the understanding of the pathophysiological links between hypertension and cognitive impairment. We emphasize the role of microvascular damage and disruptions in cerebral blood flow, which have been well-documented in previous studies as critical pathways through which hypertension exacerbates cognitive decline [15]. For instance, the neuroprotective effects of RAS blockers, as detailed in studies by Hajjar et al. [12], highlight how hypertension management can positively impact cerebrovascular health and, consequently, cognitive function. Our findings add to this body of knowledge by discussing the potential of minimizing blood pressure variability, as shown in the study of de Heus et al. [8], to stabilize cerebral blood flow and prevent cognitive deterioration.

Our review has provided new insights into the role of blood pressure variability in cognitive decline, suggesting that this factor could be a significant target for future interventions [16]. Studies such as those by de Heus et al. [16] have shown that greater variability in blood pressure is associated with more severe cognitive and functional decline in Alzheimer's patients. Managing this variability may therefore offer a promising avenue to stabilize cognitive function over time [17].

Additionally, the review highlights the complex implications of intensive versus standard blood pressure control. While intensive control aims to reduce hypertension-related risks significantly, findings from Nasrallah et al. [9] and Rapp et al. [10] suggest that such approaches might have unintended consequences on cognitive health, including greater hippocampal volume loss and a decline in processing speed. These mixed results underscore the need for tailored blood pressure management strategies that consider individual patient profiles and the potential cognitive side effects of aggressive hypertension treatment.

Our systematic review offers critical implications for both clinical treatment guidelines and public health strategies aimed at managing hypertension, particularly in populations at risk for cognitive decline and Alzheimer's disease. The nuanced findings from our review, particularly regarding the impact of intensive blood pressure control and blood pressure variability on cognitive outcomes, could inform updates to current hypertension management guidelines [18]. For elderly populations or those at risk for cognitive decline, a more personalized approach to blood pressure control might be necessary. Guidelines could recommend moderate control targets and regular monitoring to minimize blood pressure variability, which has been associated with worse cognitive outcomes. This approach would balance the need to manage hypertension effectively while reducing the risk of potential cognitive side effects associated with intensive blood pressure management [19].

Based on our findings, public health initiatives could focus on broader screening and education programs. Community-wide screening programs for hypertension in older adults could be implemented to identify individuals at risk early, allowing for timely intervention. Additionally, targeted educational programs on lifestyle modification, which has been shown to positively affect both blood pressure and cognitive health, could be developed [20]. These programs should emphasize the importance of diet, physical activity, and weight management in controlling hypertension and potentially slowing cognitive decline. Collaborations with local healthcare providers and community centers could enhance the reach and effectiveness of these interventions [21].

This study, while comprehensive, is not without limitations. Potential biases in selected studies, such as those stemming from small sample sizes or short follow-up periods, may affect the conclusions. Additionally, the variability in methods used across studies to assess cognitive decline could impact the consistency of findings. The generalizability of the results is also limited, as studies predominantly involve specific populations under controlled settings.

Future research should focus on conducting longitudinal studies that can more firmly establish causality between hypertension management and cognitive outcomes. These studies should aim to follow diverse populations over extended periods to observe the long-term effects of blood pressure control on cognitive health [22]. Moreover, clinical trials testing new interventions for blood pressure management, particularly those that explore the impact of minimizing blood pressure variability, are needed. These trials should also consider cognitive health as a primary endpoint, which would provide more targeted data to refine treatment guidelines for populations at risk of cognitive decline [23].

## Conclusions

This systematic review has critically examined the relationship between hypertension management and cognitive decline, including Alzheimer's disease, underscoring the importance of effective blood pressure control. Our findings reveal that while intensive blood pressure management may protect against cognitive impairments, it also carries potential risks, such as adverse effects on the hippocampus. Furthermore, stabilizing blood pressure variability emerges as a key factor in mitigating cognitive decline. Given the growing prevalence of both hypertension and dementia, our review advocates for a nuanced approach to hypertension management that balances cardiovascular health with cognitive outcomes. Future research should focus on longitudinal studies to refine these management strategies, enhancing treatment guidelines to better address the interconnected risks of hypertension and cognitive decline, thereby improving overall patient outcomes.
